# Identifying distinct phenotypes of patients with juvenile systemic lupus erythematosus: results from a cluster analysis by the Egyptian college of rheumatology (ECR) study group

**DOI:** 10.1186/s12887-024-05137-8

**Published:** 2024-10-25

**Authors:** Nevin Hammam, Tamer A Gheita, Ali Bakhiet, Mohamed Bakry Mahmoud, Rasha El Owaidy, Hend Abdel Nabi, Ahmed M Elsaman, Iman Khalifa, Abeer M Nour ElDin Abd ElBaky, Faten Ismail, Eman Hassan, Rawhya R El Shereef, Iman I El-Gazzar, Abdelhfeez Moshrif, Noha M Khalil, Marwa A Amer, Hanan M Fathy, Nancy Abdel Salam, Mervat I Abd Elazeem, Osman Hammam, Hanan M Fathi, Samar Tharwat

**Affiliations:** 1 Rheumatology Department, Faculty of Medicine, Assuit University, Assuit, Egypt; 2 Rheumatology Department, Faculty of Medicine, Cairo University, Cairo, Egypt; 3 Computer Science Department, Higher Institute of Computer Science and Information Systems, Culture and Science City, Giza, Egypt; 4 Pediatric Allergy, Immunology and Rheumatology Unit, Children’s Hospital, Ain Shams University, Cairo, Egypt; 5 Pediatrics Department, Rheumatology and Nephrology Unit, Tanta University, Gharbia, Egypt; 6 Rheumatology Department, Faculty of Medicine, Sohag University, Sohag, Egypt; 7 Pediatrics Unit, Helwan University, Cairo, Egypt; 8 Pediatrics Department, Medical Research and Clinical Studies Institute, National Research Centre (NRC), Cairo, Egypt; 9 Rheumatology Department, Faculty of Medicine, Minia University, Minia, Egypt; 10 Internal Medicine Department, Rheumatology Unit, Faculty of Medicine, Alexandria University, Alexandria, Egypt; 11 Rheumatology Department, Faculty of Medicine, Al-Azhar University, Assuit, Egypt; 12 Internal Medicine Department, Rheumatology Unit, Faculty of Medicine, Cairo University, Cairo, Egypt; 13 Rheumatology Department, Faculty of Medicine, Alexandria University, Alexandria, Egypt; 14 Pediatrics Nephrology Unit, Alexandria University, Alexandria, Egypt; 15 Rheumatology Department, Faculty of Medicine, Beni-Suef University, Beni-Suef, Egypt; 16 Department of Rheumatology and Rehabilitation, Faculty of Medicine, New Valley University, New Valley, Egypt; 17 Rheumatology Department, Faculty of Medicine, Fayoum University, Fayoum, Egypt; 18 Internal Medicine, Rheumatology Unit, Mansoura University, Dakahlia, Egypt

**Keywords:** Cluster analysis, Juvenile systemic lupus erythematosus, Machine learning

## Abstract

Juvenile systemic lupus erythematosus (J-SLE) is a complex, heterogeneous disease affecting multiple organs. However, the classification of its subgroups is still debated. Therefore, we investigated the aggregated clinical features in patients with J-SLE using cluster analysis. Methods: Patients (≤ 16 years) diagnosed using the Systemic Lupus International Collaborating Clinics (SLICC) classification criteria were identified from the clinical database of the Egyptian College of Rheumatology (ECR) SLE study group. Demographic data, clinical characteristics, laboratory features, and current therapies were selected. A cluster analysis was performed to identify different clinical phenotypes. Results: Overall, 404 patients, of whom 355 (87.9%) were female, had a mean age at diagnosis of 11.2 years and a mean disease duration of 2.3 years. We identified four distinct subsets of patients. Patients in cluster 1 (*n* = 103, 25.5%) were characterized predominantly by mucocutaneous and neurologic manifestations. Patients in cluster 2 (*n* = 101, 25%) were more likely to have arthritis and pulmonary manifestations. Cluster 3 (*n* = 71, 17.6%) had the lowest prevalence of arthritis and lupus nephritis (LN), indicative of mild disease intensity. Patients in cluster 4 (*n* = 129, 31.9%) have the highest frequency of arthritis, vasculitis, and LN. Cluster 1 and 4 patients had the highest disease activity index score and were less likely to use low-dose aspirin (LDA). The SLE damage index was comparable across clusters. Conclusions: Four identified J-SLE clusters express distinct clinical phenotypes. Attention should be paid to including LDA in the therapeutic regimen for J-SLE. Further work is needed to replicate and clarify the phenotype patterns in J-SLE.

**Supplementary Information:**

The online version contains supplementary material available at 10.1186/s12887-024-05137-8.

**What is Know**:

- Juvenile SLE is a heterogenous autoimmune disease with an unpredicted course and associated with an unmet need for targeted therapy.

**What is New**:

- The current study identified four distinct clinical groups in juvenile SLE using unsupervised cluster analysis.
- J-SLE group with the highest disease activity score was less likely to use prophylactic low-dose aspirin.
- Feature-driven cluster analysis using routine clinical setting data leads to the identification of aggressive J-SLE disease groups.

## Introduction

Juvenile systemic lupus erythematosus (J-SLE) is a multisystem autoimmune disease that manifests before the 18th birthday [1]. The incidence of J-SLE ranges from 0.4 to 0.9 cases per 100,000 children per year, making it a rare disease [2]. It accounts for 20% of all cases of SLE [3], and it is characterized by a greater frequency of atypical manifestations in addition to a more severe presentation and evolution. As a result, the morbidity and mortality rates associated with J-SLE are substantially higher than those associated with adult-onset disease [3–5]. In J-SLE, major organ involvement occurs more frequently than in adult-onset SLE and can include renal, neuropsychological, hematological, mucocutaneous, and cardiopulmonary manifestations [1].

The unpredictable nature of J-SLE presents significant challenges in patient care [6]. Additionally, no standardized care strategy for J-SLE exists [7]. Treatment modalities for J-SLE are extrapolated from those used for adult SLE. Randomized controlled studies in J-SLE are scarce [8]. The majority of the treatment options that are currently accessible are not targeted, and they have the potential to cause substantial side effects and toxicity, especially in age groups that are more vulnerable, such as children [1, 9]. Nevertheless, despite treatment, severe J-SLE causes early organ damage and unsatisfactory outcomes for many patients [10].

Data driven cluster analysis (CA) divides similar subjects into clusters, and being one of the unsupervised machine learning techniques, it can be used when groups are not known ahead of time [11]. Stratifying patients logically would enhance disease understanding and management. A stratification of patients with similar traits assists in the design of more targeted and effective management plans.

In J-SLE, the identification of subgroups distinguished by shared clinical and/or serologic features [12] may make it possible to better characterize the disease and may motivate targeted therapies towards the implementation of precision medicine. Previous studies [13, 14] have been conducted to identify subsets of patients with adult SLE and distinct disease patterns. Clustering based on an autoantibody profile is one of them, and it has been the subject of earlier research [15, 16]. Identification of patient clusters by clinical features and associated autoantibody profile may potentially be useful for disease prediction in daily clinical practice. However, identification of J-SLE clusters based on autoantibodies has been scarcely carried out [17]. Therefore, the aim of this study was to use CA to identify different clinical phenotypes in a large cohort of patients with J-SLE from a national data, and compare clinical and laboratory features between clusters.

## Materials and methods

### Study design and study population

This is a cross-sectional study involving a national, multicenter cohort. The study was carried out by members of the Egyptian Colleague of Rheumatology (ECR) with the involvement of specialized rheumatology departments and centers representing 13 governorates across Egypt during 2018. Patients were uniformly and broadly distributed across the country in order to eliminate any possibility of selection bias. In order to minimize missing data, all investigators were encouraged to carry out a census of their SLE patients and to fill in any missing data. Specific details on ECR-SLE study group recruitment, inclusion/exclusion criteria, and data collection procedures can be found in a prior publication [18]. All patients diagnosed with SLE before their 16th birthday by fulfilling at least four items of the 2012 Systemic Lupus International Collaborating Clinics Classification Criteria for Systemic Lupus Erythematosus (SLICC) [19] who attended any of the involved rheumatology clinics were included. From the start, patients with other rheumatic or autoimmune diseases were excluded. The study was approved by the local ethical committee of Mansoura University (R.23.09.2328). The study was carried out in accordance with the principles outlined in the Declaration of Helsinki [20]. Prior to enrollment in the study, all of the patients and/or their parents were provided with sufficient information regarding the work, and they gave their informed consent to participate.

### Demographic, clinical and laboratory data

Sociodemographic data, including age and gender, were collected from the participants. In addition, clinical data was gathered, such as the patient’s age when they were first diagnosed with lupus and the duration since the SLE diagnosis. Questions were also asked regarding the presence or absence of diabetes mellitus or hypertension. A detailed medical history and current clinical evaluation were recorded. For each patient, current disease activity and overall disease damage were calculated using the SLE disease activity index (SLEDAI) [21] and the Systemic Lupus International Collaborating Clinics/American College of Rheumatology (SLICC/ACR) Damage Index (SLICC/ACR DI) score [22]. Lupus anticoagulant and anti-cardiolipin IgG antibodies were measured using enzyme-linked immunosorbent assay (ELISA) to target antiphospholipid antibodies.

Each patient had a blood sample drawn from them on the days of their clinical examination, and an automated analyzer was used to evaluate the results of laboratory tests. The laboratory tests consisted of a complete blood count, erythrocyte sedimentation rate (ESR), serum creatinine level, serum level of complements 3 and 4 as well as antinuclear antibody (ANA) and anti-double-stranded deoxyribonucleic acid antibody (anti ds-DNA) positivity.

### Therapeutic data

The current medications use, including glucocorticoids, antimalarials, cyclophosphamide, azathioprine, cyclosporine A, mycophenolate mofetil, methotrexate, and low-dose aspirin (LDA) were reported.

### Renal pathological data

In patients with biopsy proven lupus nephritis (LN), the LN class was recorded. The renal biopsy specimens were evaluated using light microscopy (LM), and LN was classified based on International Society of Nephrology and Renal Pathology Society (ISN/RPS) criteria [23].

### Statistical analysis

Cluster analysis was performed using the K-means algorithm to group patients with similar profiles together. Clustering analysis was performed with no imputation on missing values. For continuous data, the results are shown as means with standard deviation (SD); between-group comparisons were performed using one-way analysis of variance (ANOVA). Categorical or dichotomous variables are expressed as frequencies and percentages and were compared using the chi-squared test. For statistical analysis, the significance threshold was set at *P* < 0.05. Stata statistical software version 15 (Stata-Corp), and the Python language (Ver.3.7.12) were used for the analysis of the data.

## Results

A total of 404 patients with J-SLE were eligible for the analysis. The mean (SD) age was 13.24 (2.42) years, and 355 (87.87%) were females. The mean (SD) age at disease diagnosis was 11.19 (2.55) years. Systemic hypertension accounts for 10.14% of cases, whereas diabetes mellitus only accounts for 2.97%. The majority of patients were positive for ANA and anti ds-DNA (96.29% and 82.42%, respectively). Other clinical characteristics are illustrated in Table 1.

**Table 1:** Demographics, clinical and laboratory characteristics of patients with juvenile SLE

Characteristic	All *n* = 404	Cluster 1 *n* = 103 (25.50)	Cluster 2 *n* = 101 (25.00)	Cluster 3 *n* = 71 (17.57)	Cluster 4 *n* = 129 (31.93)	
Age, years, mean ± SD	13.24 (2.42)	13.38 (2.34)	13.19 (2.52)	12.72 (2.68)	13.45 (2.22)	
Sex, female, n (%)	355 (87.87)	88 (85.43)	94 (39.07)	60 (84.51)	113 (87.60)	
Age at diagnosis, years, mean ± SD	11.19 (2.55)	11.08 (2.52)	11.57 (2.59)	11.01 (2.86)	11.07 (2.35)	
Disease duration, years, mean ± SD	2.29 (1.52)	2.39 (1.50)	2.22 (1.76)	2.02 (1.35)	2.42 (1.43)	
SLEDAI score, median (IQR)	12.0 (5.5, 17.5)	13.46 (8, 24)	7.0 (5, 13.4)^a^	6.0 (2, 16)^a^	14 (8, 22)^b, c^	
SLICC DI score, mean ± SD	0.28 (0.71)	0.33 (0.80)	0.32 (0.75)	0.13 (0.37)	0.30 (0.74)	
Hypertension, n (%)	41 (10.14)	19 (18.45)	1 (0.99)^a^	2 (2.82)^a^	19 (14.73)^b, c^	
Diabetes mellitus, n (%)	12 (2.97)	6 (5.82)	2 (1.98)	3 (4.22)	1 (0.77)	
***Laboratory parameters***						
Hemoglobin, g/dl, median (IQR)	9.48 (8, 11)	9.0 (7.6, 10.6)	10.1 (9.5, 12)^a^	10.4 (9.1, 11.5)^b^	8.8 (7.2, 9.7)^b, c^	
WBCs, x10^3^/mm^3^, median (IQR)	5.8 (3.5, 7.6)	4.8 (3.2, 7.6)	6.0 (4, 7.6)	6.0 (4.8, 7.9)	5.0 (3.2, 7.2)	
PLT, x10^3^/mm^3^, median (IQR)	207.0 (139.5, 290)	180.0 (128, 230)	219.2 (154, 316)^a^	230 (164, 300)	181 (117, 242)	
ESR, mm/1st h, median (IQR)	69.2 (40, 100)	69.2 (50, 98)	65.0 (40, 85)	60.0 (28, 70)	70 (50, 101)^c^	
Creatinine, mg/dl, median (IQR)	0.77 (0.50, 0.9)	0.88 (0.5, 1.13)	0.64 (0.41, 0.88)^a^	0.80 (0.50, 0.88)^a^	0.70 (0.50, 0.91)	
ANA positivity, n (%)	389 (96.29)	98 (95.14)	96 (95.05)	71 (100.0)	124 (96.12)	
Anti- ds-DNA positivity, mean ± SD	333 (82.42)	87 (84.46)	81 (80.20)	53 (74.65)	112 (86.82)	
Antiphospholipid positivity, n (%) (*n* = 304) ^#^	67 (22.0)	15 (4.9)	24 (7.9)	13 (4.3)	15 (4.9)	
Low Complement 3, mean ± SD	243 (60.15)	88 (85.43)	30 (29.70)^a^	16 (22.53)^a^	109 (84.49)^b, c^	
Low Complement 4, mean ± SD	176 (43.65)	67 (65.04)	12 (11.88)^a^	12 (16.90)^a^	85 (65.89)^b, c^	
***Treatment***,*** current use***						
Glucocorticoid use, n (%)	362 (89.60)	103 (100.0)	78 (77.23)^a^	53 (74.64)^a^	128 (99.22)^b, c^	
Hydroxychloroquine, n (%)	289 (71.53)	89 (86.41)	50 (49.50)^a^	49 (69.01)^a, b^	101 (78.29)^b^	
Cyclophosphamide, n (%)	108 (26.73)	40 (38.83)	5 (4.95)^a^	2 (2.82)^a^	61 (47.29)^b, c^	
Azathioprine, n (%)	119 (29.45)	31 (30.10)	33 (32.67)	26 (36.62)	29 (22.48)	
Cyclosporine A, n (%)	9 (2.23)	3 (2.91)	1 (0.99)	2 (2.82)	3 (2.32)	
Mycophenolate mofetil, n (%)	147 (36.39)	47 (45.63)	14 (13.86)^a^	6 (8.45)^a^	80 (62.01)^a, b,c^	
Methotrexate, n (%)	20 (4.95)	8 (7.77)	1 (0.99)	3 (4.22)	8 (6.20)	
Low dose aspirin, n (%)	36 (8.91)	6 (1.48)	19 (18.81)^a^	4 (5.63)^b^	7 (5.43)^b^	
Renal biopsy class (*n* = 146)	146 (36.14)	34 (33.01)	38 (37.62)	10 (14.08)	64 (49.61)	
I-II, n (%)	57 (39.04)	11 (32.35)	19 (50.00)	7 (70.00)	20 (31.25)	
III-IV, n (%)	71 (48.63)	21 (61.76)	14 (36.84)	2 (20.00)^a, b^	34 (53.12)^a, c^	
V-VI, n (%)	18 (12.33)	2 (5.88)	5 (13.16)	1 (10.00)	10 (15.62)	

SD: standard deviation; IQR: inter-quartile range; SLE: systemic lupus erythematosus; SLEDAI: SLE Disease Activity Index; SLICC DI: Systemic Lupus Erythematosus International Collaborating Clinics Damage Index; WBCs: white blood cells, PLT: platelets, ESR: erythrocyte sedimentation rate; ANA: antinuclear antibody; and anti-dsDNA: anti–double-stranded deoxyribonucleic acid antibody

^#^Antiphospholipid positivity represents both lupus anticoagulant and anti-cardiolipin IgG antibodies

^a^ Significantly different from cluster 1, ^b^ Significantly different from cluster 2, ^c^ Significantly different from cluster 3

The most frequently prescribed immunosuppressive drugs were glucocorticoids (89.60%), antimalarials (71.53%), followed by mycophenolate mofetil (36.39%), cyclophosphamide (26.73%), and azathioprine (29.45%), while methotrexate (4.95%) and cyclosporine A (2.23%) were the least frequently prescribed medications. Only 36 patients (8.91%) received a prescription for LDA.

Of the study patients with the available data, 146 (36%) had biopsy proven nephritis. Classes III-IV were the most frequently encountered classes, accounting for 71 (48.63%) patients, followed by classes I-II in 57 (39.04%), while classes V-VI were discovered in only 18 (12.33%) patients.

### Clustering of the patients

**Fig. 1:**
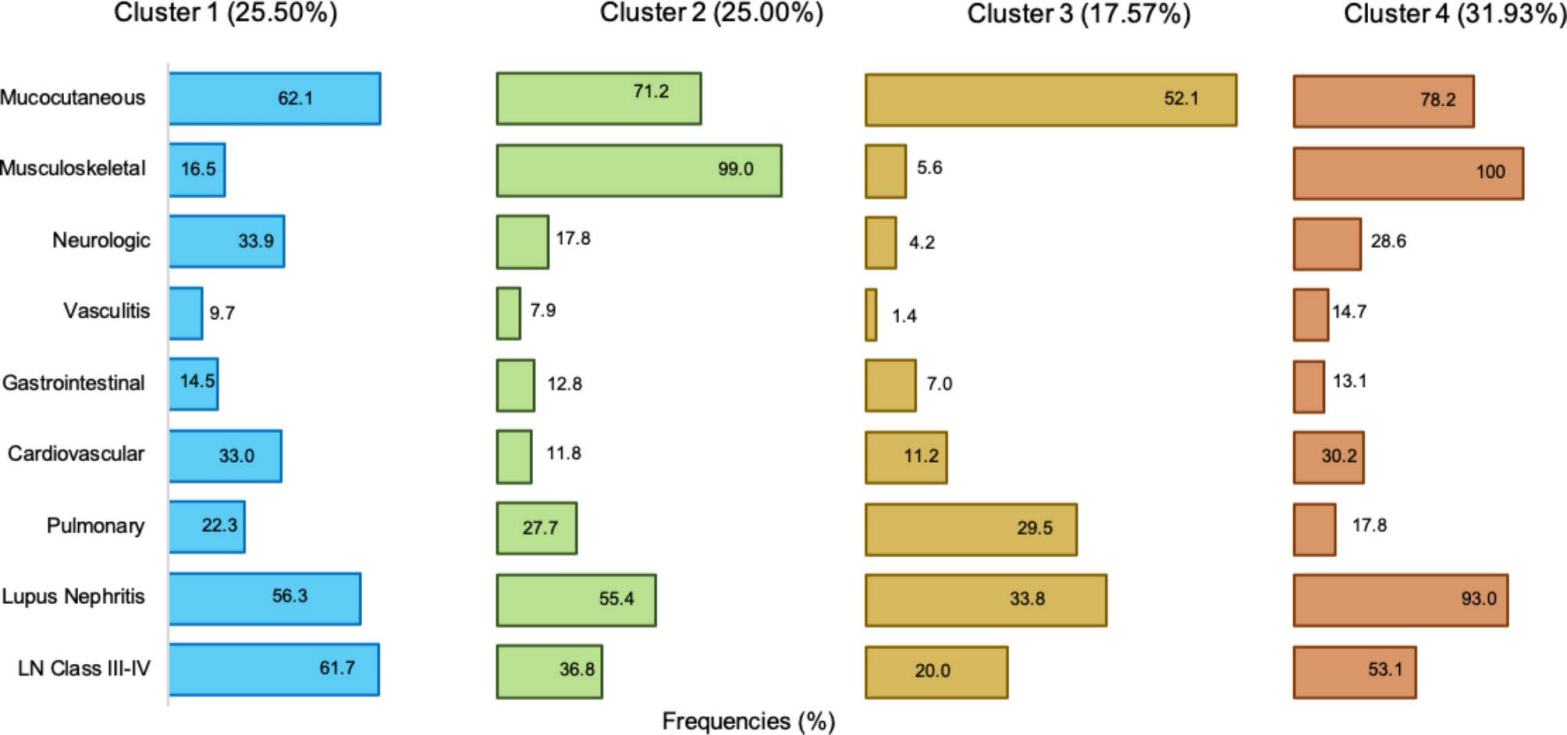
Demonstrate the frequencies of different clinical manifestations of four cluster groups of Juvenile SLE patients identified using unsupervised cluster analysis (*N* = 404)

Our data set was divided into four distinct groups, as demonstrated in Table 1; Fig. 1. There were no undifferentiated cases that didn’t fit into any cluster, nor no cases that fit into at least 2 clusters. Patients in Cluster 1 (C1) consisted of 103 patients (25.5%,) and were distinguished primarily by the presence of mucocutaneous manifestations, as well as neurologic manifestations, which occurred in 62.1% and 33.9% of C1 patients, respectively.

Patients in Cluster 2 (C2) included 101 patients (25.0%) who were more likely to have musculoskeletal and pulmonary manifestations. Musculoskeletal manifestations occur in 99.0% of C2 patients compared to 16.5% in C1 and 5.6% in C3 patients. The frequency of pulmonary manifestations was higher in patients with C3 (29.5%) than in patients with C1 (22.3%), C2 (27.7%) and C4 (17.8%) (Supplementary Table 1).

Cluster 3 (C3) included 71 patients (17.6%) with the lowest frequency of musculoskeletal manifestations and LN, suggesting mild disease severity. Meanwhile, LN was present in 33.8% of C3, compared to 56.3% in C1, 55.4% in C2, and 93.0% in C4.

Patients in Cluster 4 (C4) included 129 patients (31.9%) with the highest frequency of musculoskeletal manifestations, vasculitis, and renal involvement.

The severity of the disease was different across clusters; patients in clusters 1 and 4 had the highest SLEDAI score and were significantly less likely to use LDA. However, SLICC/ACR DI scores were comparable across clusters (*p* > 0.05).

## Discussion

In this large national J-SLE cohort, we employed one of the statistical approaches of CA to identify groups of J-SLE patients with similar patterns and to describe the clinical differences between these groups. The application of CA made it possible to characterize four J-SLE phenotype groups. Patients in C1 and C4 had the highest disease activity index score and were significantly less likely to use LDA. SLE damage index scores were comparable across clusters. Feature-driven CA leads to the identification of more aggressive disease groups. Although there are studies available that focus on clustering in adult-onset SLE populations, this is, as far as we are aware, the first large national study to be conducted within the context of J-SLE.

In fact, the dissection of disease heterogeneity is currently regarded as one of the most significant contemporary challenges facing the management of SLE [24]. Incomplete understanding of the underlying biological mechanisms of disease and the heterogeneity of the disease across poorly defined clusters of individuals are key road obstacles in the development of new lupus treatments [25, 26]. The utilization of clinical clustering in the context of heterogeneous diseases serves the purpose of identifying distinct subsets of diseases. This approach holds potential for forecasting disease patterns, as well as anticipating the severity of the disease and the associated complications in various organs [27].

The majority of studies that attempted to cluster SLE focused on the adult-onset form of the disease. In an early attempt to evaluate autoantibody clusters and their correlations with clinical characteristics and organ damage accrual in adult-onset SLE patients, *To and Petri* conducted a study on 1,357 adult SLE patients and identified three distinct autoantibody clusters [16]. In another study that was carried out in the United States of America on 198 patients with adult-onset SLE, the researchers used thorough molecular phenotyping and machine learning clustering to address the issue of heterogeneity in SLE, and the results revealed seven distinct clusters of these patients [28]. In 2019, *Lanata and coauthors* devised a stepwise multi-omics technique for identifying SLE patient subgroups across a multi-ethnic cohort. Using this method, they identified three patient clusters that varied according to the severity of the disease [29]. On the other hand, *Ahn et al.* identified three distinct phenotypic clusters based on the damage index among a cohort of 1,130 individuals diagnosed with SLE. Patients in group C1 had the least amount of tissue damage. C2 was distinguished by the pervasiveness of renal and ocular damage, whereas C3 was dominated by neuropsychiatric and musculoskeletal damage [30].

There is a paucity of data pertaining to disease clusters in J-SLE patients. *Jurencak et al.* attempted to identify J-SLE patients with comparable autoantibody patterns and ascertained their clinical associations using CA. They carried out a single-center cohort analysis with 169 individuals, and CA indicated three different autoantibody clusters. C1 had mild disease with infrequent major organ involvement, as opposed to cluster 2, which had the highest frequency of nephritis, renal failure, serositis, and hemolytic anemia, or cluster 3, which had frequent neuropsychiatric disease and nephritis [31]. In 2019, *Torrente-Segarra et al.* conducted a multicenter, descriptive, cross-sectional study on a cohort of 345 J-SLE patients from the Spanish Society of Rheumatology Lupus Registry in order to detect clusters of damage presentation [17]. Three damage clusters were identified: C1 had a significantly lower number of individuals with damage; C2 had renal damage in 60% of patients, which was significantly more than C1 and C3, in addition to increased ocular, cardiovascular, and gonadal damage, while C3 was the only group with musculoskeletal damage. In 2022, a retrospective chart review of 53 patients with J-SLE and biopsy-confirmed LN was undertaken in order to identify autoantibody clusters indicative of end stage renal disease (ESRD) in a biopsy-proven juvenile LN. CA demonstrated the highest frequency of ESRD in the group, with LN defined by anti-Ro/SSA and anti-dsDNA co-positivity [32].

J-SLE is challenging to treat, and the treatment differs from patient to patient and from center to center [2]. In the current study, the most prescribed immunosuppressive medicines were glucocorticoids (89.60%) and antimalarials (71.53%), followed by mycophenolate mofetil, azathioprine, and cyclophosphamide. This is consistent with a longitudinal study from the United Kingdom in which 349 patients with JSLE were included. MMF was the most commonly utilized first-line immunomodulating drug, followed by azathioprine and MTX [2]. The use of cyclophosphamide, a cytotoxic drug, raises major concerns, particularly when it comes to children and young adults with J-SLE. Cyclophosphamide is often utilized primarily in JSLE patients with more severe organ involvement [33, 34].

The cornerstone of SLE-directed therapy is the prescription of antimalarials, which are intended for long-term use and are nearly universally prescribed for all newly diagnosed patients [35]. Nevertheless, we found that not all our patients with J-SLE were taking antimalarials. This can be attributed to the lack of adherence to the treatment regimen. Adherence to treatment is particularly relevant in J-SLE population [36]. The reported adherence rate for antimalarials is 49%, as determined by pharmacy refill data [37].

In the present sample, we observed that patients in clusters with the highest disease activity index scores (C1 and C4) were significantly less likely to take LDA than patients in other clusters. In fact, LDA is reported to be safe, useful for thromboprophylaxis in antiphospholipid syndrome, and can considerably decrease the incidence of atherosclerotic cardiovascular disease events in SLE patients [32, 38]. Beyond cardiovascular disease, LDA has been related to a decreased risk of overall mortality [39]. However, there is currently no data available on the association between the use of LDA and lupus disease activity. Due to the cross-sectional nature of the study that can’t examine the causal inference and the complexes of the variables, the direct relationship between LDA and SLICC score can’t be drawn from the study.

The renal outcomes of classes I and II of lupus nephritis are not well known, however it is generally assumed that their renal prognosis is favorable [40]. In the current study, it was interesting that classes I-II were frequently encountered classes, accounting for 57 (39.04%) patients with biopsy proven LN. The distribution of renal classes in the present study is not fully understood, and the evolution of renal outcome of these patients was not analyzed. However, it may be related to the different demographic traits. Also, it can be explained by the fact that general pediatricians in Egypt are typically the ones who begin the process of following up with SLE children and they have a low threshold for asking for a kidney biopsy. In a prospective evaluation of lupus nephritis in Egyptian children over a 16-year, a renal biopsy was done in 132 patients and LN class II represents 23% [41]. A comparable frequency of childhood LN class II was reported in an observational experience from another developing country [42]. From the other side, a metanalysis evaluating the prevalence of biopsy-proven LN [43] identified class IV as the most prevalent and the one associated with the highest risk of progression to end stage renal disease.

Patients with SLE are less likely to sustain damage while in a state of long-term remission or lupus low disease activity state. Calcineurin inhibitors (cyclosporine, tacrolimus, and voclosporin) appear to be a viable treatment option for J-SLE patients, particularly those with LN [44]. The SHARE project has yielded recommendations for the diagnosis, management, and treatment of J-SLE, which are based on the most reliable evidence and expert opinion [40]. Thus, the achievement of a low disease activity state in lupus is a desirable objective for treatment [45]. However, we found no link between activity and damage indices in our cohort.

Antimalarials are prescribed nearly universally for SLE patients. They are inexpensive, especially compared with treatments used more recently in SLE patients, and also have a good overall tolerability profile [46]. Their utilization has been linked to decreased disease activity and damage [47]. These medications have protective effects on a variety of clinical aspects, including cardiovascular, gastrointestinal, renal, or hematological manifestations [48]. In this regard, we observed that the use of antimalarials was considerably lower in C2 patients, which may be related to the higher incidence of arthritis and pulmonary manifestations in this cluster.

CA is a useful statistical method that can be used to create subgroups on the basis of the features of the data. It develops the clusters that are the most statistically valid by minimizing the distances within clusters and maximizing the distances among clusters. As a result, the subgroups are determined only by the data themselves, without the involvement of the researcher [49]. In recent years, there has been an increase in interest in the practicability of cluster analysis in pediatric rheumatology for disentangling the heterogeneity of disease and locating subgroups [50, 51].

There are some strengths and limitations in this study. The major strength of our study was the large number of patients derived from many centres across the whole country and the strictly defined inclusion criteria, which allowed for well-categorized J-SLE groups. Besides, the rigorous way that the clinical data were collected to make sure that it was comprehensive representing the disease’s heterogeneity. On the other hand, there were limitations: due to the cross-sectional nature of the study that can’t examine the causal inference and the complexes of the variables, and the direct relationship between LDA and SLICC score can’t be drawn from the study. Lack of the current steroid dose is another limitation of the study, which need to be examined in the future work. We were not able to analyze the delay in disease diagnosis since the time of the disease onset was not available. Because of economic issues, none of the study participants were using biologic therapy. CA is an exploratory analysis that is used to identify subsets of cases if the grouping is not previously known. Therefore, it does not make any distinction between dependent and independent variables. CA does not provide an explanation of why these clusters exist, and techniques for determining the reliability and validity of clusters have not yet been developed.

## Conclusions

In conclusion, the CA in a large national cohort using clinical features divided the patients with J-SLE into four clusters. These subtypes express different phenotypes with diverse disease activity. The use of LDA appears to be associated with a mild severity form of the disease. Our preliminary findings of the subgroup pattern require further replication to identify the similarity in other cohorts and even among other ethnicities. This analysis may help clinicians identify the disease subtypes accurately and arrange for personalized treatment.

## Electronic Supplementary Material

Below is the link to the electronic supplementary material.

Supplementary Material 1

## Data Availability

The corresponding author will provide the dataset and/or the used code upon request for a reasonable purpose.

## Abbreviations

ANA: Antinuclear antibody
Anti-ds DNA: Anti-double-stranded deoxyribonucleic acid antibodies
ANOVA: One-way analysis of variance
C: Cluster
CA: Cluster analysis
ECR: Egyptian College of Rheumatology
ISN/RPS: International Society of Nephrology and Renal Pathology Society
J-SLE: Juvenile systemic lupus erythematosus
LDA: Low-dose aspirin
LM: Light microscopy
LN: Lupus nephritis
SD: Standard deviation
SLICC: Systemic Lupus International Collaborating Clinics Classification Criteria for Systemic Lupus Erythematosus
SLEDAI: SLE disease activity index
SLICC/ACR DI: Systemic Lupus International Collaborating Clinics/American College of Rheumatology (SLICC/ACR) Damage Index
ESRD: End stage renal disease
