# Supplementary material for: Identifying distinct phenotypes of patients with juvenile systemic lupus erythematosus: results from a cluster analysis by the Egyptian college of rheumatology (ECR) study group
Source: BMC Pediatr. 2024 Oct 25;24:679. doi: 10.1186/s12887-024-05137-8 (PMC11515332; doi:10.1186/s12887-024-05137-8)
Supplement: Supplementary file 1 — Supplementary Material 1 [file 12887_2024_5137_MOESM1_ESM.docx]

Supplementary Table 1: Clinical characteristics of patients with JSLE

| Clinical characteristics | Cluster 1  n = 103  (25.50) | Cluster 2  n = 101  (25.00) | Cluster 3  n = 71  (17.57) | Cluster 4  n = 129  (31.93) | *P^1^* | *P^2^* | *P^3^* | *P^4^* | *P^5^* | *P^6^* |
| --- | --- | --- | --- | --- | --- | --- | --- | --- | --- | --- |
| Mucocutaneous | 64 (62.14) | 72 (71.29) | 37 (52.11) | 101 (78.29) | 0.487 | 0.492 | 0.04 | 0.037 | 0.66 | 0.001 |
| Musculoskeletal | 17 (16.5) | 100 (99.01) | 4  (5.63) | 129 (100) | <0.001 | 0.007 | <0.001 | <0.001 | 0.986 | <0.001 |
| Neurologic | 35 (33.98) | 18 (17.82) | 3  (4.23) | 37 (28.68) | 0.026 | <0.001 | 0.762 | 0.142 | 0.192 | <0.001 |
| Vasculitis | 10 (9.71) | 8  (7.92) | 1  (1.41) | 19 (14.73) | 0.971 | 0.248 | 0.556 | 0.468 | 0.29 | 0.011 |
| Gastrointestinal | 15 (14.56) | 13 (12.87) | 5  (7.04) | 17 (13.18) | 0.983 | 0.452 | 0.989 | 0.665 | 1 | 0.59 |
| Cardiovascular | 34 (33.01) | 12 (11.88) | 8 (11.27) | 39 (30.23) | 0.002 | 0.004 | 0.956 | 1 | 0.005 | 0.01 |
| Pulmonary | 23 (22.33) | 28 (27.72) | 21 (29.58) | 23 (17.83) | 0.8 | 0.684 | 0.853 | 0.992 | 0.295 | 0.24 |
| Lupus Nephritis | 58 (56.31) | 56 (55.45) | 24 (33.8) | 120 (93.02) | 0.999 | 0.004 | <0.001 | 0.007 | <0.001 | <0.001 |
| LN Class III-IV | 21 (61.76) | 14 (36.84) | 2  (20) | 34 (53.12) | 0.988 | 0.034 | 0.021 | 0.015 | 0.057 | <0.001 |

^1^Cluster 2 vs cluster 1, ^2^Cluster 3 vs cluster 1, ^3^Cluster 4 vs cluster 1, ^4^Cluster 3 vs cluster 2, ^5^Cluster 4 vs cluster 2, and ^6^Cluster 4 vs cluster 3.

Mucocutaneous manifestations include malar rash, discoid rash, photosensitivity, oral ulcer; Musculoskeletal manifestations include arthritis and myositis; Neurologic manifestations include depression, concentration problem, chorea, seizure, lupus headache, and peripheral neuropathy; Gastrointestinal manifestations include abdominal pain, hepatomegaly, elevated transaminase, and autoimmune hepatitis; Cardiovascular manifestations include pericarditis, myocarditis, endocarditis, and valvular insufficiency; Pulmonary manifestations include pleurisy/pleural effusion, and pulmonary infiltrates; and Lupus Nephritis according to the 2018 revised classification of the International Society of Nephrology and the Renal Pathology Society.
